# Dual‐Zinc Electrode Electrochemical Desalination

**DOI:** 10.1002/cssc.202000188

**Published:** 2020-03-19

**Authors:** Jinhong Dai, Jian Wang, Xianhua Hou, Qiang Ru, Qingyu He, Pattarachai Srimuk, Volker Presser, Fuming Chen

**Affiliations:** ^1^ Guangdong Provincial Key Laboratory of Quantum Engineering and Quantum Materials Guangdong Engineering Technology Research Center of Efficient Green Energy and Environment Protection Materials School of Physics and Telecommunication Engineering South China Normal University Guangzhou 510006 P.R. China; ^2^ INM - Leibniz Institute for New Materials Campus D2 2 66123 Saarbrücken Germany; ^3^ Department of Materials Science & Engineering Saarland University Campus D2 2 66123 Saarbrücken Germany

**Keywords:** deionization, dual-zinc electrode, electrochemical desalination, redox mediators, water remediation

## Abstract

Continuous and low‐energy desalination technologies are in high demand to enable sustainable water remediation. Our work introduces a continuous desalination process based on the redox reaction of a dual‐zinc electrode. The system consists of two zinc foils as redox electrodes with flowing ZnCl_2_ electrolyte, concentrated and diluted salt streams with three anion‐ and cation‐exchange membranes (AEM and CEM) separated configuration (AEM|CEM|AEM). If a constant current is applied, the negative zinc electrode is oxidized, and electrons are released to the external circuit, whereas the positive zinc electrode is reduced, causing salt removal in the dilution stream. The results showed that brackish water can be directly desalted to 380.6 ppm during a continuous batch‐mode process. The energy consumption can be as low as 35.30 kJ mol^−1^ at a current density of 0.25 mA cm^−2^, which is comparable to reverse osmosis. In addition, the dual‐zinc electrode electrochemical desalination demonstrates excellent rate performance, reversibility, and batch cyclability through electrode exchange regeneration. Our research provides a route for continuous low‐energy desalination based on metal redox mediators.

## Graphical abstract

**Continuous desalination**! A novel dual‐zinc electrode electrochemical desalination process allows continuous desalination at low energy consumption. Brackish water is directly desalted from 2000 to 380.6 ppm during a continuous batch‐mode process. The energy consumption is as low as 35.30 kJ mol^−1^ at a current density of 0.25 mA cm^−2^, which is comparable to reverse osmosis.

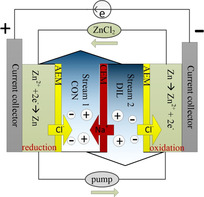

## Introduction

Advanced water remediation requires technologies that provide energy efficiency and continuous operation. Present‐day large‐scale desalination methods, such as reverse osmosis (RO), thermal distillation, and electrodialysis, can achieve these goals and have been practically applied in many cases.^1^, ^2^ However, the capital investments, operating costs, and energy consumption are still high. Capacitive deionization (CDI) has been proposed as an energy‐saving and economic deionization approach.^3^, ^4^, ^5^, ^6^ However, the removal capacity of a conventional CDI device remains very low owing to the limited ion electrosorption capacity of carbon electrode materials. A higher desalination performance can be obtained, even at high molar strength, when charge–transfer (faradaic) materials are added to carbon or completely replace the carbon.^1^, ^7^, ^8^, ^9^, ^10^ Although continuous salt removal has been achieved by the use of suspension electrodes, they are prone to clogging, and the low mass loading per volume limits the device‐based desalination performance metrics.^11^, ^12^, ^13^, ^14^

Dual‐ion faradaic deionization is a promising new concept for electrochemical desalination. This technology can be used to achieve an ion capture capacity that is up to six times higher than conventional CDI.^15^, ^16^, ^17^ Various deionization designs based on the faradaic electrode reaction have been proposed, including rocking‐chair desalination,^18^, ^19^, ^20^ redox‐flow battery desalination,^21^, ^22^, ^23^ and some novel design desalination.^1^, ^24^, ^25^, ^26^, ^27^, ^28^, ^29^ These desalination devices operate by the use of individual capture/release of ions by two separate electrode materials. The removal capacity is limited by the intrinsic specific capacity of the electrode materials, and cell operation still alternates between half‐cycles of desalination (charging) and regeneration (discharging), which makes continuous operation impossible.^1^, ^30^ Semicontinuous operation could be enabled by alternating the two channels in a manner synchronized with the direction of the applied current. Once saturated, an inverse current is applied to achieve regeneration.^19^ Electrochemical desalination has also been reported for cells based on redox electrolytes, such as K_3_[Fe(CN)_6_]/K_4_[Fe(CN)_6_], Zn/ZnCl_2_, I_3_^−^/I^−^, and Cu/CuSO_4_.^29^, ^31^, ^32^, ^33^ These aforementioned redox‐electrolyte systems do not yet provide continuous operation owing to the separated electrode design.^22^, ^28^ The integration of a continuous, energy‐saving, economic, and easy‐operation desalination technology is a key asset for the development of advanced desalination devices.

Here, we present a continuous and energy‐economic desalination method based on a dual‐zinc electrode redox electrochemical process. The zinc ion recirculates between the flowing positive and negative electrodes and is oxidized at the negative electrode and reduced at the positive chamber during the charge applied with constant current, resulting in a continuous desalination process with unlimited salt removal capacity. The performance of the salt removal rate, reversibility cycling, and batch cyclability through electrode exchange regeneration were investigated. The change of the salt content was monitored by a conductivity meter. This energy‐economic continuous desalination method will make a significant contribution to future practical desalination applications.

## Experimental Section

### Materials and solution preparation

ZnCl_2_ (97 % purity) and NaCl (99 % purity) were obtained from Sigma–Aldrich and used without any further purification. Salt feed water was prepared by dissolving NaCl salt in 100 mL of deionized water to prepare 2000 ppm salt feed. Two 25 mL NaCl salt solutions were drawn as the test feeds. The anodic and cathodic electrolyte was prepared by adding 1.5 mmol ZnCl_2_ electrolyte in 100 mL deionized water at room temperature. To remove the oxygen dissolved in the solutions, pure nitrogen gas was used to purge the aqueous media prior to the tests. The anion‐/cation‐exchange membrane (AEM/CEM, standard grade) was ordered from Tokuyama.

### Experimental setup

**Figure 1:**
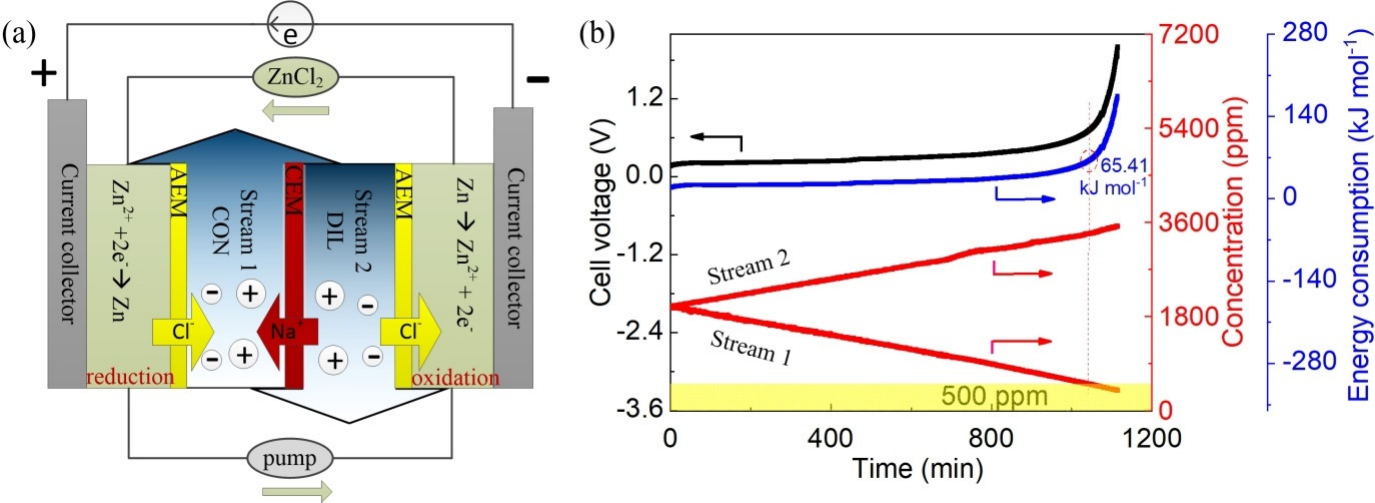
(a) Schematic diagram of continuous desalination with a dual‐zinc electrode. (b) Continuous desalination performance and corresponding instant energy consumption with 15 mm ZnCl_2_ electrolyte under 0.25 mA cm^−2^ current density.

A schematic diagram of the desalination system based on redox reactions is shown in Figure 1 a. As shown in Figure S1 in the Supporting Information, the device consisted of a ZnCl_2_ negative stream electrolyte, two middle salt streams (concentrated and diluted), and the ZnCl_2_ positive stream electrolyte. Two zinc foils (2×2 cm) with 0.15–0.25 mm thickness were contacted by platinum wires as the current collectors in the ZnCl_2_ electrolyte. The foils were polished with sandpaper and washed with acetone, diluted hydrochloric acid, and deionized water before usage. A CEM was placed between salt reservoirs 1 and 2, and a total of two (one on each side) AEMs was placed between the salt reservoir and the positive/negative reservoir. The ZnCl_2_ electrolyte (100 mL solution with 15 mm ZnCl_2_ electrolyte) flowed at 11.52 mL min^−1^ through the positive and negative reservoir connected to each other by a soft tube, then circled back to the container. The contents of the two salt channels (25 mL each with 2000 ppm NaCl salt) were circulated separately. Each compartment chamber of salt feed 1 and 2 or electrolyte had a cylinder dimension of 1.0 cm height and 3.5 cm diameter. All sheets were made from acrylic plates.

### Electrochemical desalination

The desalination tests were performed by using a battery analyzer (Neware). The salt concentration in stream 1 and 2 was recorded online by using conductivity meters (eDAQ, EPU357). The flow rates were set to 11.52 mL min^−1^ by using a peristaltic pump. Before the desalination test, the cell was kept circulating for 1 h without an applied voltage. A current density of 0.25 mA cm^−2^ was applied for most of the desalination tests. To study the rate influence, we varied the current density from 0.125 to 2 mA cm^−2^. The initial salt concentration was 2000 ppm. The desalination device was charged for 100 min at 0.125 mA cm^−2^, held for 50 min at every subsequent electrical current density, and returned to 0.125 mA cm^−2^ for 100 min. Inductively coupled plasma optical emission spectroscopy (ICP‐OES) was used to analyze the zinc ion concentration in the two salt streams after desalination to quantify the leak of zinc ions. We also studied the influence of a NaCl salt concentration of 1000, 2000, 3000, and 4000 ppm and ZnCl_2_ electrolyte concentration of 5, 10, 15, and 50 mm. The charge/discharge cycling was realized by controlling the alternating current density at ±0.25 mA cm^−2^. The batch cyclability was performed by electrode exchange regeneration and refreshment of the salt feed.

### Calculation of performance metrics

The salt removal rate *v* [μg cm^−2^ min^−1^], which describes the desalination speed, can be calculated by the following equation [Eq. (1)]:^34^, ^35^

(1) $$\nu =\left(\frac{\Delta c}{\Delta t}\times V\right)/A$$

in which $\frac{\Delta c}{\Delta t}$ is the salt concentration change per minute [Δppm min^−1^], *V* is the volume of the salt stream [mL], and the active area of the cell *A* is 4 cm^2^ (zinc foil area).

The charge efficiency *Γ* [%], which is defined as the percentage of salt removal to electrons used, is an important performance parameter for electrochemical desalination. It can be obtained by the following equation [Eq. (2)]:^36^

(2) $$\Gamma =\frac{\left(c_{t}-c_{0}\right)\times V\times 10^{-3}/M_{NaCl}}{I\times \left(t_{t}-t_{0}\right)/F}$$

in which *c*_t_ and *c*_0_ are the NaCl salt concentrations [ppm] at *t*_t_ and *t*_0_ time [s], respectively. *V* is the volume [L] of NaCl salt solution, *M*_NaCl_ is the molar mass of NaCl (58.44 g mol^−1^), *I* is the applied current [A], and F is the Faraday constant (96 485 C mol^−1^).

The energy consumption *Ē* [kJ mol^−1^] can be calculated as follows [Eq. (3)]:

(3) $$\bar{E}=\frac{3.6\Delta E}{\left[\left(c_{0}-c_{t}\right)\times \frac{V}{10^{6}\times M_{NaCl}}\right]}$$

in which Δ*E* is the total energy consumption [Wh] during the charging process, *c*_0_ and *c_t_* are the initial NaCl salt concentration and at time *t* [ppm], respectively, *V* is the volume of the salt stream [mL], and *M*_NaCl_ is the molar mass of NaCl (58.44 g mol^−1^).

## Results and Discussion

### Continuous desalination with the dual‐zinc electrode

A schematic diagram of desalination is displayed in Figure 1 a with flowing ZnCl_2_ as the redox electrolyte between a positive and negative zinc electrode. The detailed components are shown in Figure S1 in the Supporting Information. Four reservoir streams are recirculated using a multichannel peristaltic pump. The constant current is supplied by a battery analyzer.

As a first baseline, we studied the general redox behavior of zinc by half‐cell measurements (three‐electrode setup) using cyclic voltammetry (CV), as shown in Figure S2 in the Supporting Information. Only one pair of redox peaks was observed: The reduction peak was located at −1.11 V vs. Ag/AgCl whereas the corresponding oxidation peak was at −0.98 V vs. Ag/AgCl. During the cathodic current, the Zn^2+^ was electrochemically deposited on the surface of the zinc foil. As for the oxidation process, the process appeared to be highly reversible. When a constant was current applied (Figure 1 a), zinc was oxidized in the negative chamber, and zinc ions were reduced at the positive side during charging. With the addition of ionexchange membranes, chloride ions in stream 2 were captured by the negative reservoir and released to stream 1 through the AEM at the negative side. At the same time, sodium ions in stream 2 were transported via the middle CEM to stream 1. These processes ensured charge neutrality within the electrolyte chambers. Overall, salt in stream 2 was continuously removed to stream 1, as shown in Figure 1 b. The NaCl concentration of stream 2 decreased from 2000 to 380.6 ppm (81 % salt removal), yielding a desalination rate of 9.0 μg cm^−2^ min^−1^ and a charge efficiency of up to 99.1 %.

The proposed technology shares similarities with reverse electrodialysis. The key concept of this approach is the utilization of zinc redox reactions for charge transfer across the membranes to restrict the increase of the voltage. Therefore, low energy consumption could be achieved in this work. The operating voltage plateau was maintained at less than 0.5 V during the initial 960 min test, as shown in Figure S3 in the Supporting Information. The zinc redox potential allows for energy‐efficient desalination. However, the eventual voltage increase was owing to ion depletion following Nernst behavior. The instant energy consumption during the salt removal is shown in Figure 1 b. The initial instant energy consumption was 19.0 kJ mol^−1^. As time increased, more energy was consumed owing to the slight voltage rise. At the point of 500 ppm freshwater product in the diluted stream 2, 65.41 kJ should be consumed per 1 mol of removed salt. The addition of membranes can increase the resistance of the system and, thereby, lower the kinetic response. As shown in Figure S4 in the Supporting Information, the solution resistance was 77.7 Ω for ZnCl_2_ electrolyte; however, the three stacked ion‐exchanged membranes possess a resistance of 151.1 Ω. To further confirm the solution resistance change during the desalination, electrochemical impedance spectroscopy (EIS) measurements were performed at different times, as shown Figure S5 in the Supporting Information. The solution resistance increased owing to the ion depletion in the diluted stream.

To quantify the leak of zinc ions, we used ICP‐OES to determine the concentration of zinc ions in the two salt streams after desalination. The results showed that the zinc concentrations in the diluted stream and the concentrated stream were 0.1 and 1.6 ppm, respectively, well below the limit of 5 ppm in drinking water stated by the World Health Organization. An even lower loss of zinc is expected if improved ion‐exchange membranes that exhibit better permselectivity are used.^29^

### Impact of current density on desalination performance

**Figure 2:**
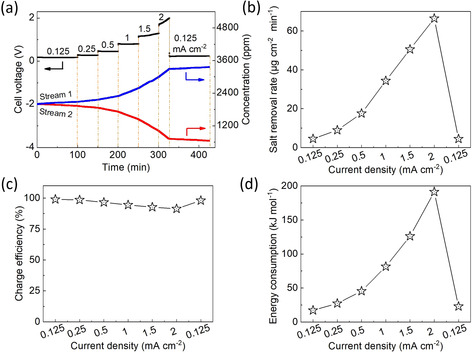
(a) Zn/Zn^2+^ redox‐mediated desalination at various current densities (0.125 mA cm^−2^ for 100 min, 0.25 mA cm^−2^ for 50 min, 0.5 mA cm^−2^ for 50 min, 1 mA cm^−2^ for 50 min, 1.5 mA cm^−2^ for 50 min, 2 mA cm^−2^ for 50 min, and 0.125 mA cm^−2^ for 100 min) and corresponding performance of (b) salt removal rate, (c) charge efficiency, and (d) energy consumption at various current densities.

The direct correlation between the electric charge and ion concentration to balance the latter allows us to control the salt removal rate by controlling the current density. The variations of voltage and salt content at different currents densities, which varies from 0.125 to 2 mA cm^−2^, are shown in Figure 2. The voltage plateau increased with increasing current density. Considering the decomposition of water, we set the upper limit of the voltage to 2 V. When a current density of 2 mA cm^−2^ was applied, the voltage dramatically increased to 2 V. The salt removal rate (Figure S6 and Table S1 in the Supporting Information) increased with increasing current density, as demonstrated in Figure 2 b, indicating a fast desalination process at a high current density. The desalination rate was 4.5, 9.0, 17.6, 34.4, 50.6, and 66.4 μg cm^−2^ min^−1^ at the corresponding current density of 0.125, 0.25, 0.5, 1, 1.5, and 2 mA cm^−2^, respectively. The increase in the rate of salt concentration of stream 1 aligned with the salt increase measured in stream 2 (Table S1 in the Supporting Information). The charge efficiency was maintained at more than 91.4 % at the various current densities (Figure 2 c). These results were consistent with the previous reports.^37^, ^38^

The energy consumption at various current densities is displayed in Figure 2 d. At a current density of 0.125 mA cm^−2^, the average energy consumption was relatively low, 19.0 kJ mol^−1^, owing to the low operation voltage plateau, which was comparable to values reported for RO,^39^, ^40^, ^41^, ^42^, ^43^ as shown in Figure S7 in the Supporting Information.. To achieve a low energy consumption of the zinc redox system, it is important to limit the voltage increase. The low energy consumption was enabled by the low operation voltage plateau at 0.125 mA cm^−2^ and the afforded charge efficiency. More energy must be spent at a higher current density, namely, 27.3–191.4 kJ mol^−1^ for 0.25–2 mA cm^−2^. The curve of the energy consumption versus salt removal rate at different current densities is shown in Figure S8 in the Supporting Information. For comparison, intermittent operation of a VCl_3_/NaI flow‐battery desalination consumes approximately 10 kJ mol^−1^.^22^ Other flow battery desalination systems and desalination batteries show much higher levels for energy consumption at the same current density. For example, the metal‐free 4‐hydroxy‐2,2,6,6‐tetramethylpiperidine 1‐oxyl as redox mediator requires approximately 79–205 kJ mol^−1^ at 0.06–0.89 mA cm^−2^.^44^

### Reversibility cycling of dual‐zinc electrode redox desalination

**Figure 3:**
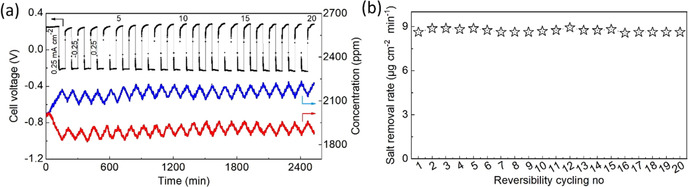
(a) Charge/discharge cycling and salt concentration change at a current density of ±0.25 mA cm^−2^, and (b) corresponding performance of the salt removal rate.

The reversible charge/discharge redox process is shown in Figure 3. The current was controlled to alternate at ±0.25 mA cm^−2^ for 1 h each step except for the initial 2 h charge process. At a current density of −0.25 mA cm^−2^ (Figure 3 a), the salt content in stream 1 was decreased whereas the opposite was observed in stream 2. The 20 charge/discharge cycles demonstrated excellent reversibility enabled by the highly reversible redox process. The corresponding salt removal rate of approximately 8.7 μg cm^−2^ min^−1^ is shown in Figure 3 b, and the corresponding energy consumption (23.5–25.9 kJ mol^−1^) and charge efficiency (95.0–98.4 %) are shown in Figure S9 in the Supporting Information. These results are consistent with the literature.^45^ The measured salt removal/release rates in streams 1 and 2 were experimentally equal (Table S2 in the Supporting Information).

### Batch cyclability through electrode exchange regeneration

**Figure 4:**
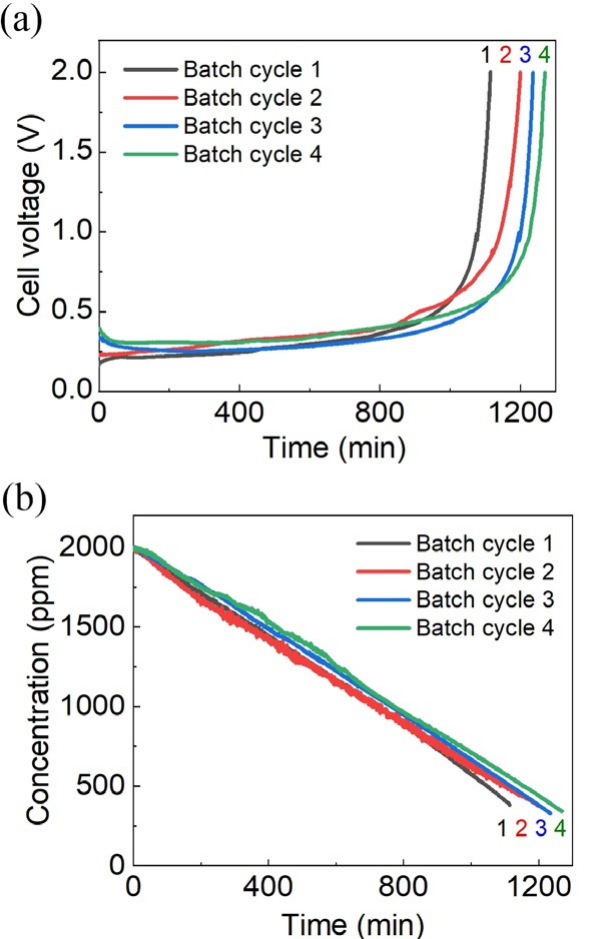
Four batch cycling tests: (a) variation of voltages and (b) salt concentration change in the diluted stream at 0.25 mA cm^−2^ current density.

In the desalination, batch cycling allows examination of the system and performance stability. Because dual‐zinc electrodes were used, batch cycling at the laboratory scale could be achieved through electrode exchange and refreshment of the salt feed. The consumed Zn from the last cycle is restored and the Zn grown from the last cycle is depleted upon subsequent cycling. The data for four batch cycling tests is presented in Figure 4 and Figure S10 in the Supporting Information. With increased batch cycling, the voltage plateau and desalination time slightly increase. The energy consumption, salt removal rate, and charge efficiency are shown in Figure S10 in the Supporting Information. For the first cycle, the energy consumption was 35 kJ mol^−1^. As the voltage plateau slightly increased in subsequent cycles, the energy consumption also increased. For example, in the 2nd, 3rd, and 4th cycle, the energy consumption gradually increased to 40, 43, and 47 kJ mol^−1^, respectively. The removal rate dropped to 8.2 μg cm^−2^ min^−1^ in the last cycle from 9.0 μg cm^−2^ min^−1^ in the initial cycle, and the charge efficiency slightly decreased to 90.1 %, as shown in Figure S10 b, c in the Supporting Information.

### Influence of salt feed concentration and electrolyte concentration

**Figure 5:**
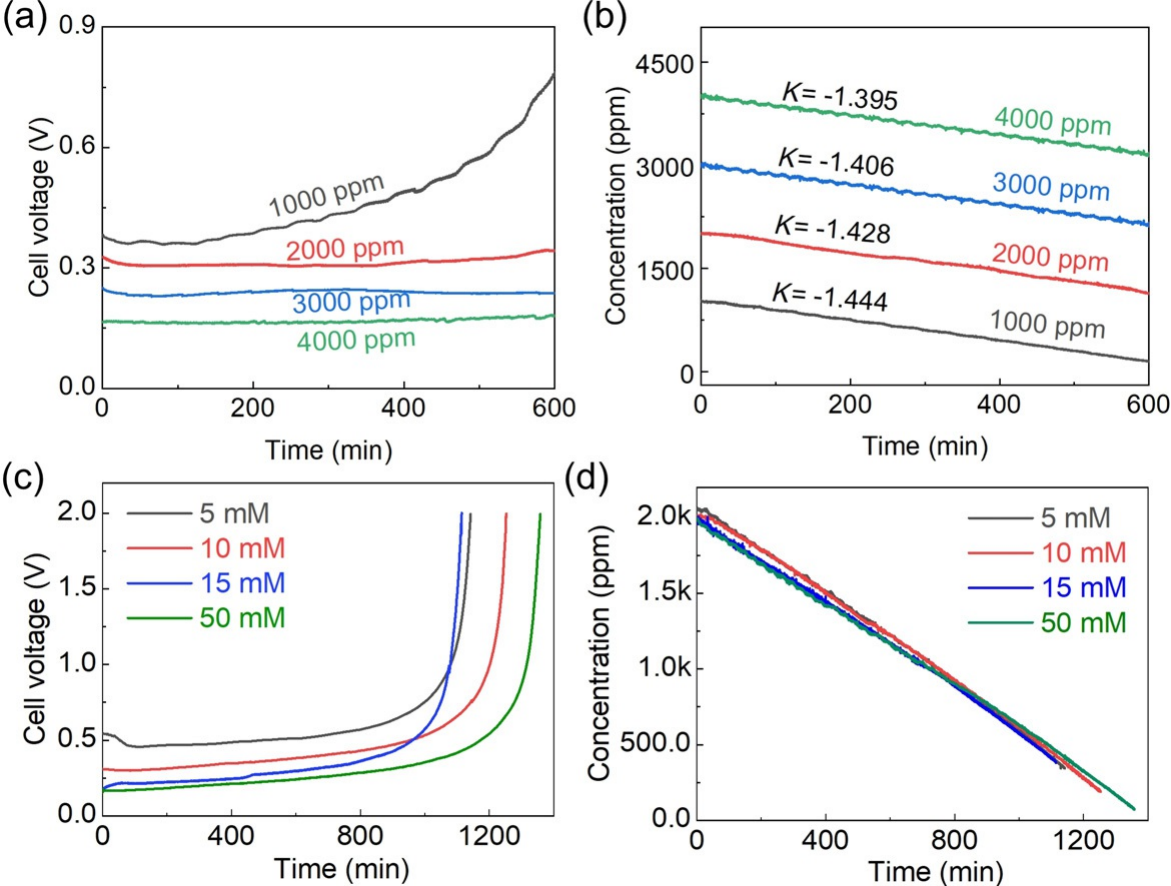
(a) Effect of salt feed concentration the voltage change, and (b) corresponding salt concentration variation in the diluted stream at 0.25 mA cm^−2^ current density. Effect of ZnCl_2_ electrolyte concentration, (c) variation curves of the voltage and (d) salt concentration in the diluted stream at different concentrations of ZnCl_2_.

The change in operating voltage in different feedwater salt concentrations is shown in Figure 5. At a high salt concentration, the polarization was low, causing a low operating voltage. Therefore, in the testing samples, the voltage platform with an initial 4000 ppm feed was the lowest. The concentration change in stream 2 at different salt concentrations is shown in Figure 5 b. A high‐concentration salt feed can result in a low operating voltage, whereas a high slope is obtained at a low salt feed solution. This may be owing to the more serious repelling action of the co‐ion in the high‐concentration conditions during the coupling desalination. This result was consistent with previous studies.^44^ The removal rate, charge efficiency, and consumption data are shown in Figure S11 in the Supporting Information.

The influence of the ZnCl_2_ electrolyte concentration was investigated by using 5, 10, 15, and 50 mm ZnCl_2_, as shown in Figure 5 c, d and Figure S12 in the Supporting Information. A higher electrolyte concentration can result in a low voltage platform. In the tested samples, the lowest operating plateau was obtained when 50 mm ZnCl_2_ electrolyte was used. The corresponding salt removal rate, charge efficiency, and energy consumption decreased with the increase of the electrolyte concentration, as demonstrated in Figure S12 in the Supporting Information. Therefore, 15 mm ZnCl_2_ was chosen for further tests in this work.

## Conclusions

A continuous and low‐energy‐consumption desalination method was developed based on a dual‐zinc electrode electrochemical redox process. The device consisted of two zinc foils, one cation‐exchange membrane (CEM), two anion‐exchange membranes (AEMs), two middle salt reservoirs (diluted and concentrated), and a circulated ZnCl_2_ electrolyte between two electrode reservoirs. The zinc foils were connected by platinum wire as the current collector. A CEM was placed between the salt reservoirs, and AEMs were placed between the salt reservoir and the positive/negative side‐reservoirs. During the charging process, zinc is oxidized in the negatively polarized chamber whereas zinc ions are reduced at the positive side, resulting in continuous salt removal as long as charge flows. The novel system can be used to continuously dilute the NaCl concentration in brackish water to a drinking water level. The energy consumption can be as low as 35 kJ mol^−1^ for the treatment of 2000 ppm NaCl feed to freshwater, which is comparable with values typically found for commercial reverse osmosis systems. In addition, the desalination performance was influenced by the current intensity, salt feed concentration, and electrolyte concentration. Therefore, the desired desalination performance could be controlled by adjusting the current, salt feed, and electrolyte. The current system also demonstrated promising charge/discharge reversibility and batch cycling through current reversal. The latter enables regeneration of the Zn redox system to continue to serve as the mechanism by which salt is removed. Such a system enables unlimited and continuous salt removal without any content change in the electrode reservoirs, which is a key asset for future desalination and renewable energy efforts. Therefore, this new method could be a breakthrough in the development of continuous desalination systems based on the redox electrochemical technique.

## Conflict of interest

*The authors declare no conflict of interest*.

## Supporting information

As a service to our authors and readers, this journal provides supporting information supplied by the authors. Such materials are peer reviewed and may be re‐organized for online delivery, but are not copy‐edited or typeset. Technical support issues arising from supporting information (other than missing files) should be addressed to the authors.

Supplementary
