# Supplementary material for: Dual‐Zinc Electrode Electrochemical Desalination
Source: ChemSusChem. 2020 Mar 19;13(10):2792–8. doi: 10.1002/cssc.202000188 (PMC7318675; doi:10.1002/cssc.202000188)
Supplement: Supplementary file 1 — Supplementary [file CSSC-13-2792-s001.pdf]

ChemSusChem

Supporting Information

Dual-Zinc Electrode Electrochemical Desalination

Jinhong Dai,[a] Jian Wang,[a] Xianhua Hou,[a] Qiang Ru,[a] Qingyu He,*[a] Pattarachai Srimuk,[b, c] Volker Presser,*[b, c] and Fuming Chen*[a]

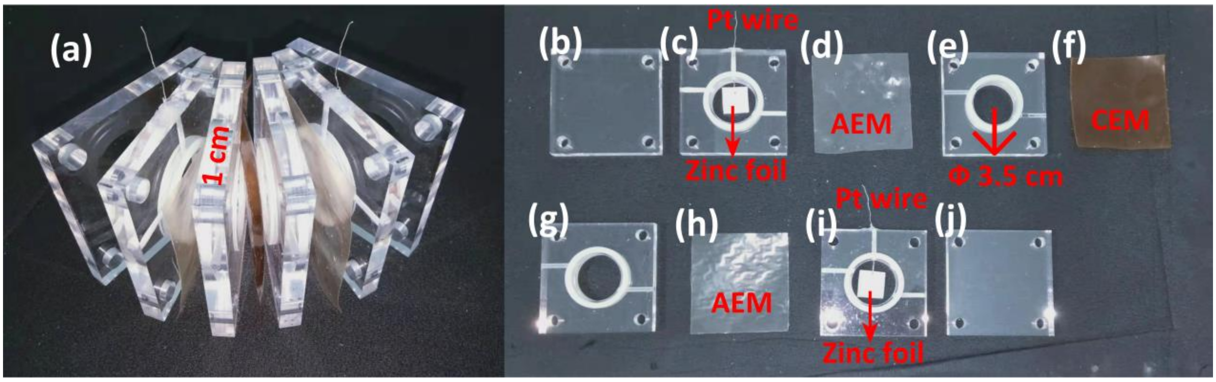

Figure S1. The compartment photograph of continuous redox electrochemical desalination, (a) the assembled structure of deionization device, and the separated parts (b-j), the device (a) can be obtained by assembling (b-j) in sequence.

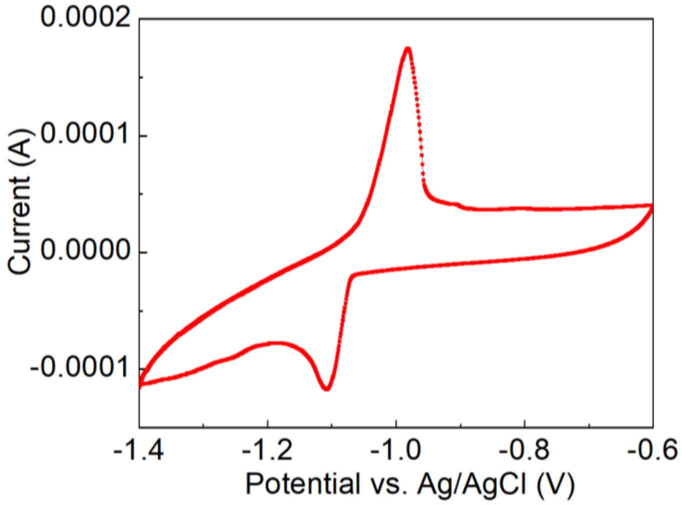

Figure S2. The three-electrode cyclic voltammogram of zinc in the $ZnCl_2$ electrolyte.

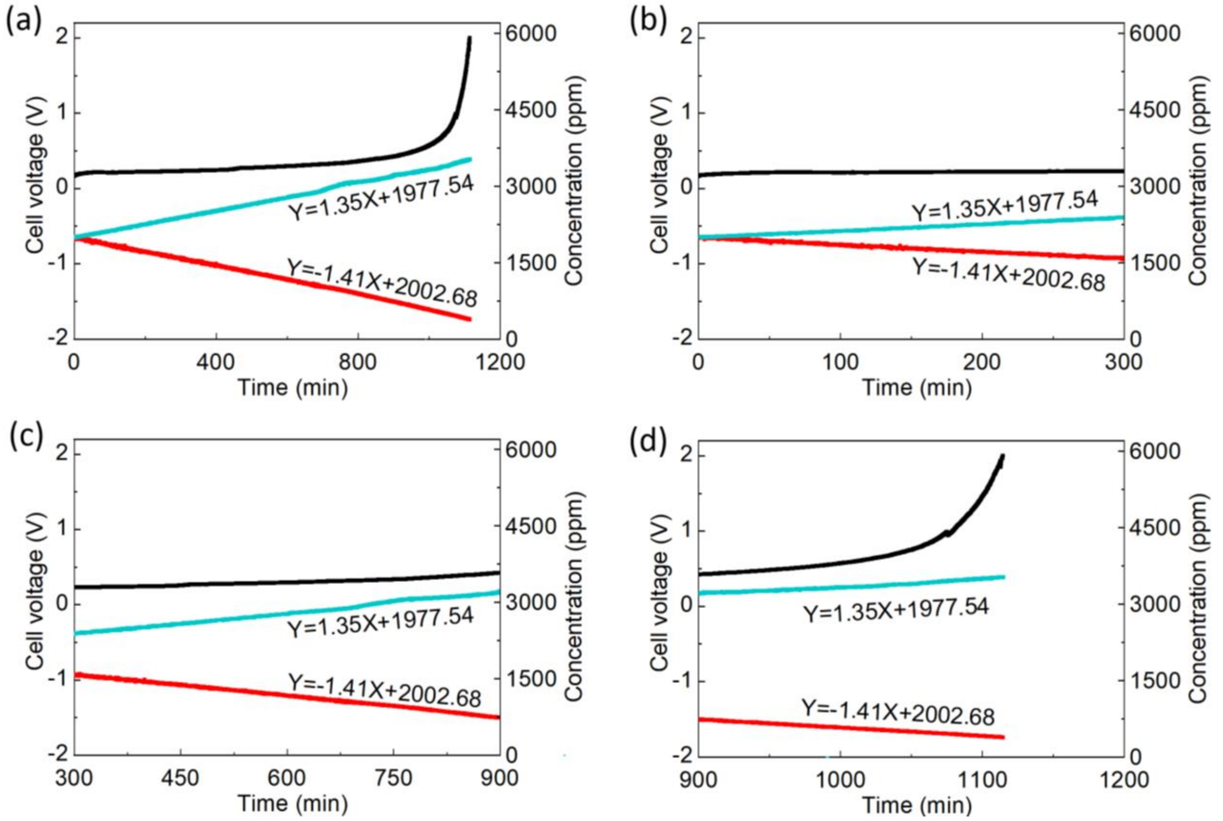

Figure S3. (a) The continuous desalination performance of dual-zinc electrode redox electrochemical process at the 0.25 mA·cm^{-2} current density, (b-d) the zoom-in section at different times.

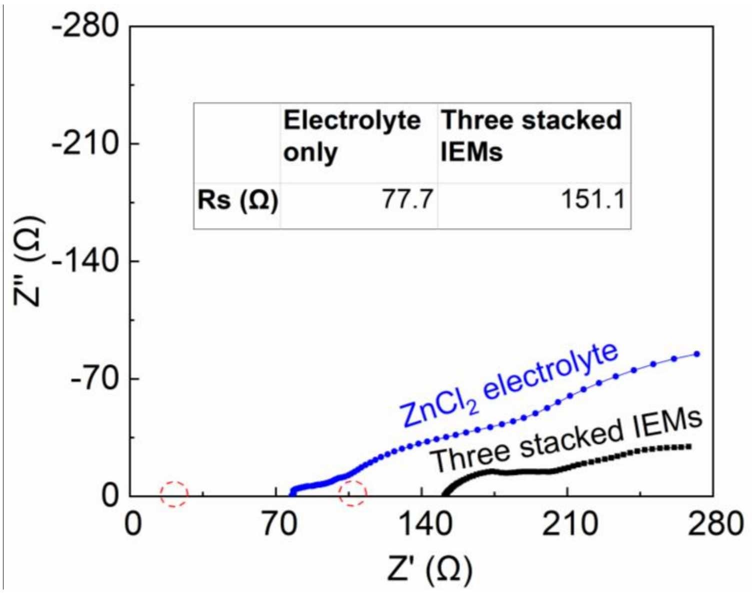

Figure S4. EIS results of electrolyte only ($ZnCl_2$ without membranes) and three stacked ion exchanged membranes, and the corresponding solution resistance (Rs)

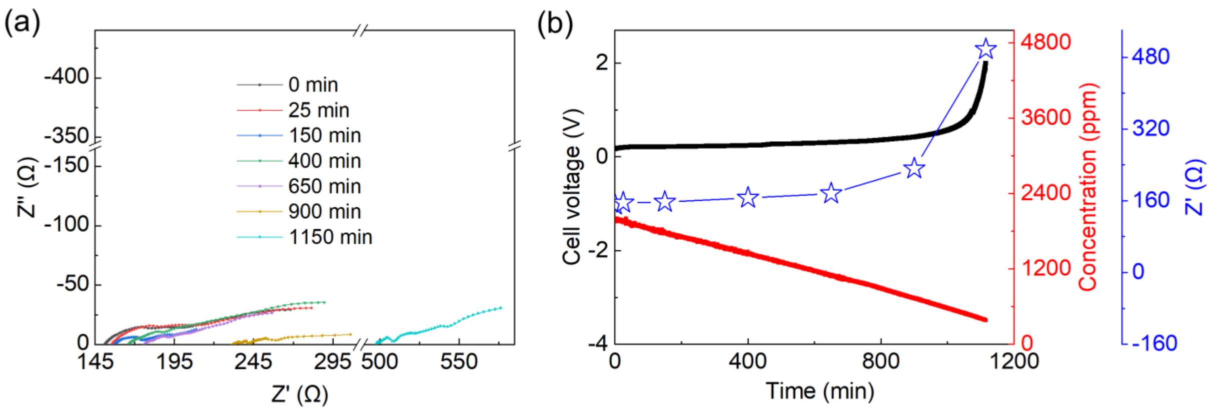

Figure S5. (a) EIS results of three stacked ion exchanged membranes during the desalination, and (b) the corresponding change of cell voltage, salt concentration in dilute stream, and solution resistance (Rs)

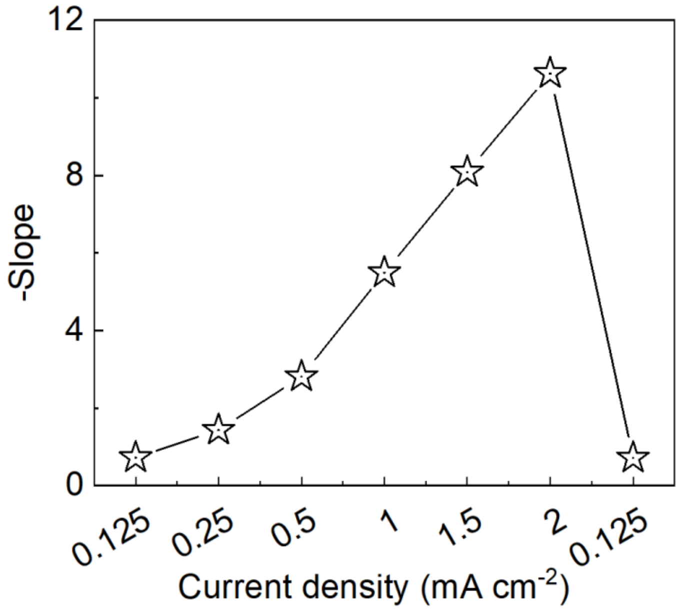

Figure S6. The negative slope at various current densities in Figure 2a

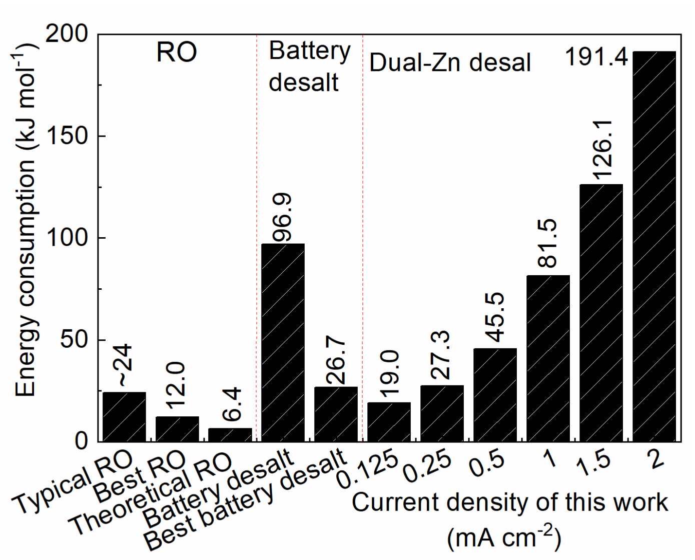

Figure S7. Comparison of energy consumption for RO, battery desalination, and the current dual-zinc desalination with a variety of current densities. The energy consumption of typical RO is around 24 kJ mol^{-1} salts while the best energy consumption achieved by RO is 12.03 kJ mol^{-1}.[1-3] The theoretical energy consumption by RO is 1.06 kWh m^{-3} at 50% water recovery (converting to 6.38 kJ mol^{-1}). Energy consumption by typical battery desalting,[4] best reported battery desalting,[5] results in the current work were obtained in 0.125-2 mA cm^{-2}.

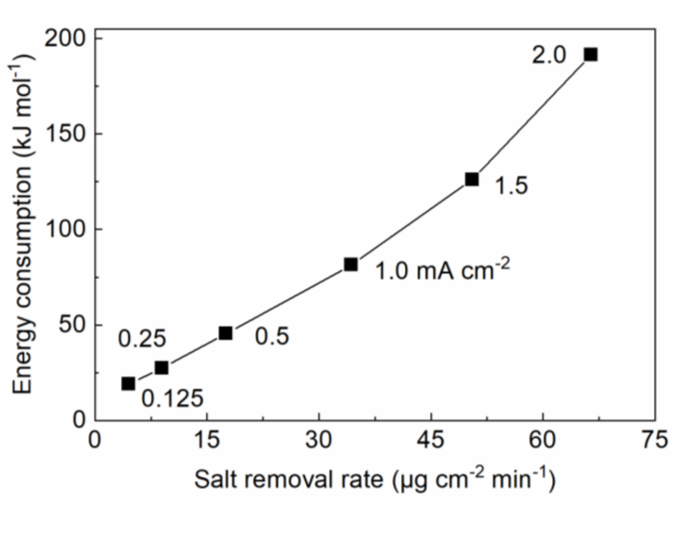

Figure S8. The curve of energy consumption vs. salt removal rate at different current densities.

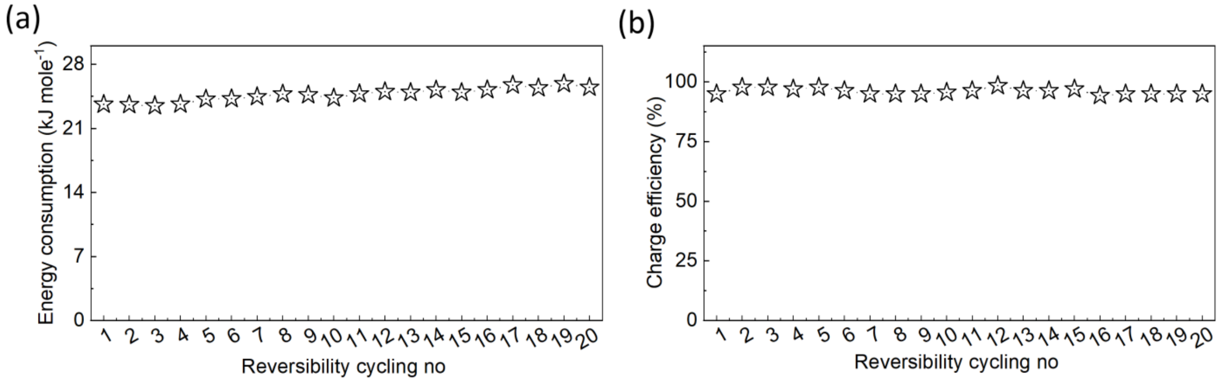

Figure S9. The energy consumption (a) and charge efficiency (b) during the reversibility cycling in Figure 3 at a current density of ±0.25 mA·cm^{-2}.

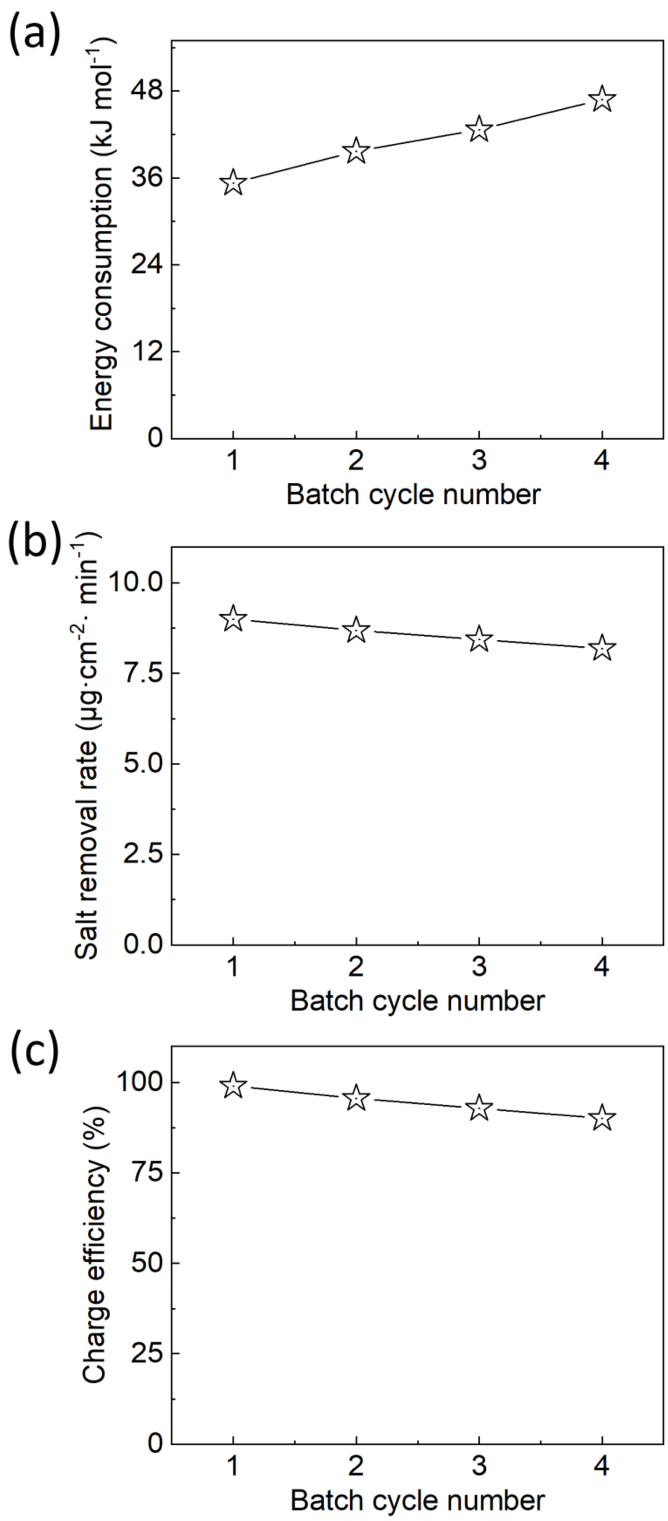

Figure S10. The energy consumption (a), salt removal rate (b), and charge efficiency (c) during the batch cycling (Figure 4).

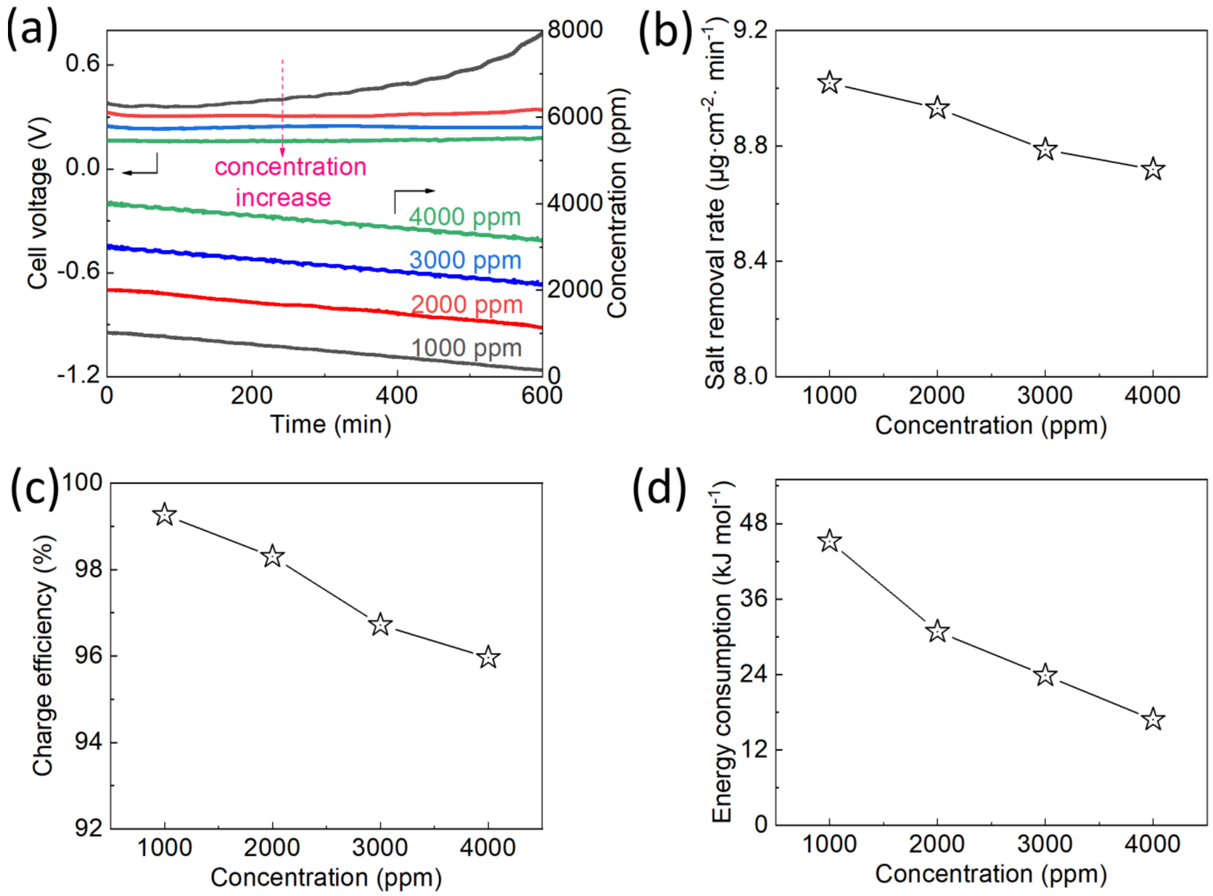

Figure S11. The influence of salt concentration, (a) the curves of voltage and the corresponding salt concentration change in the diluted stream at varied concentrations; the corresponding performance of the salt removal rate (b), charge efficiency (c), energy consumption (d).

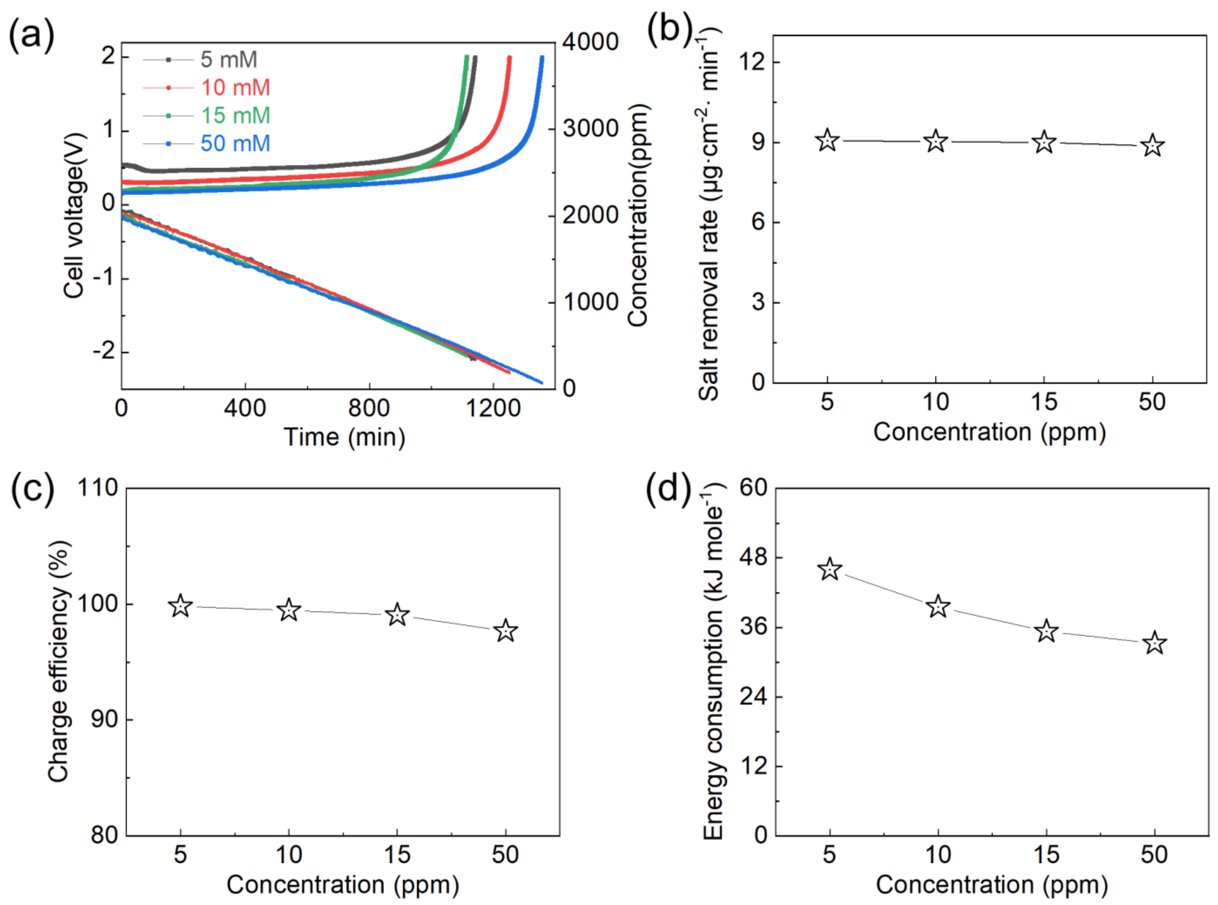

Figure S12. The influence of $ZnCl_2$ electrolyte concentration, (a) the curves of voltage and the corresponding salt concentration change in the diluted stream at varied $ZnCl_2$ electrolyte concentrations; the corresponding performance of the salt removal rate (b), charge efficiency (c), energy consumption (d).

Table S1. The comparison of the salt grown rate in stream 1 and the salt removal rate in stream 2 at different currents.

Item	Stream	Current density (mA·cm^{-2})						
		0.125	0.25	0.5	1	1.5	2	0.125
Salt concentration change rate (μg·cm^{-2}·min^{-1})	1	4.5	9.0	17.6	34.4	50.6	66.4	4.4
	2	4.9	9.8	17.0	35.0	50.0	67.1	4.6

Table S2. The comparison of salt removal/release rates in stream 1 and 2 during the reversibility cycling tests.

Item	Cycle number	Stream	
		1	2
Salt concentration change rate ($\mu g \cdot cm^{-2} \cdot min^{-1}$)	1	8.6	8.8
	2	8.9	9.0
	3	8.9	8.6
	4	8.8	8.4
	5	8.9	8.8
	6	8.8	8.9
	7	8.6	8.9
	8	8.6	8.6
	9	8.6	8.7
	10	8.7	8.5
	11	8.8	9.3
	12	8.9	8.3
	13	8.8	8.4
	14	8.8	8.4
	15	8.8	8.4
	16	8.6	8.3
	17	8.6	8.7
	18	8.6	8.8
	19	8.6	9.0
	20	8.6	8.8

References:

1. S. Choi, B. Chang, J. H. Kang, M. S. Diallo and J. W. Choi, *Journal of Membrane Science*, 2017, **541**, 580-586.
2. M. Elimelech and W. A. Phillip, *Science*, 2011, **333**, 712-717.
3. S. Lin and M. Elimelech, *Desalination*, 2015, **366**, 9-14.
4. F. Chen, Y. Huang, L. Guo, L. Sun, Y. Wang and H. Y. Yang, *Energy & Environmental Science*, 2017, **10**, 2081-2089.
5. Y. Huang, F. Chen, L. Guo, J. Zhang, T. Chen and H. Y. Yang, *Desalination*, 2019, **451**, 241-247.

1. R. Zhao, S. Porada, P. M. Biesheuvel and A. van der Wal, *Desalination*, 2013, **330**, 35-41.
2. S. Choi, B. Chang, J. H. Kang, M. S. Diallo and J. W. Choi, *Journal of Membrane Science*, 2017, **541**, 580-586.
3. M. Elimelech and W. A. Phillip, *Science*, 2011, **333**, 712-717.
4. S. Lin and M. Elimelech, *Desalination*, 2015, **366**, 9-14.
5. F. Chen, Y. Huang, L. Guo, L. Sun, Y. Wang and H. Y. Yang, *Energy & Environmental Science*, 2017, **10**, 2081-2089.
6. Y. Huang, F. Chen, L. Guo, J. Zhang, T. Chen and H. Y. Yang, *Desalination*, 2019, **451**, 241-247.
